# Rare Presentation of Small Bowel Diverticula: A Novel Case Report and Insights Into Diagnostic Dilemmas and Clinical Spectrum

**DOI:** 10.7759/cureus.79512

**Published:** 2025-02-23

**Authors:** Murad M Hamiedah, Rawan M Ayyad, Sa'ed N Alsmadi, Omar Makhamreh, Hussien R Al-Nawaiseh, Bilal Al-Bdour

**Affiliations:** 1 General Surgery, King Hussein Medical Center/Jordanian Royal Medical Services, Amman, JOR; 2 Radiotherapy, Military Cancer Center/Jordanian Royal Medical Services, Amman, JOR; 3 Hepato-pancreato-biliary Surgery, King Hussein Medical Center/Jordanian Royal Medical Services, Amman, JOR; 4 Surgical Oncology, King Hussein Medical Center/Jordanian Royal Medical Services, Amman, JOR

**Keywords:** benign pneumoperitoneum, exploration laparotomy, jejunal diverticulosis, small bowel diverticulosis, small bowel surgery

## Abstract

Small bowel diverticulosis is an uncommon condition with a broad spectrum of clinical presentations, often challenging to diagnose and manage. This report presents a 38-year-old male patient who initially presented with vague, colicky abdominal pain that acutely intensified, accompanied by pneumoperitoneum on imaging. Exploratory laparotomy revealed multiple diverticula in the jejunum and ileum, with a perforated jejunal segment, which was surgically resected. Despite subsequent conservative management, the patient continued to experience intermittent abdominal pain and recurrent pneumoperitoneum on imaging over a two-year follow-up, eventually requiring a second laparotomy for a perforated diverticulum. Postoperatively, he experienced additional complications, including incisional hernias. This case underscores the diagnostic challenges and need for individualized management in small bowel diverticulosis, where both conservative and surgical strategies may be required to balance symptom relief and prevent recurrence.

## Introduction

Small bowel diverticulosis is a rare condition, characterized by the formation of true diverticula sac-like protrusions that involve all layers of the small bowel wall, commonly found in the jejunum and ileum [1]. Unlike colonic diverticulosis, which is prevalent and often asymptomatic, small bowel diverticulosis has an incidence of only 0.1% to 2.3% and frequently presents diagnostic challenges due to its nonspecific and variable symptoms [2]. Most cases are asymptomatic or present with vague symptoms, such as intermittent abdominal pain, which can mimic a variety of gastrointestinal disorders. However, in certain cases, it can lead to severe complications including diverticulitis, perforation, or obstruction, which often necessitate surgical intervention [3].

This case report discusses the individualization of management in a 38-year-old male patient who experienced intermittent vague abdominal pain and acute and recurrent complications of small bowel diverticulosis. Despite undergoing conservative management, the patient's recurrent symptoms underscore the diagnostic challenges and both surgical and nonsurgical dilemmas of small bowel diverticulosis, particularly when recurrent imaging reveals pneumoperitoneum without clear clinical indications for intervention [4].

## Case presentation

**Figure 1:**
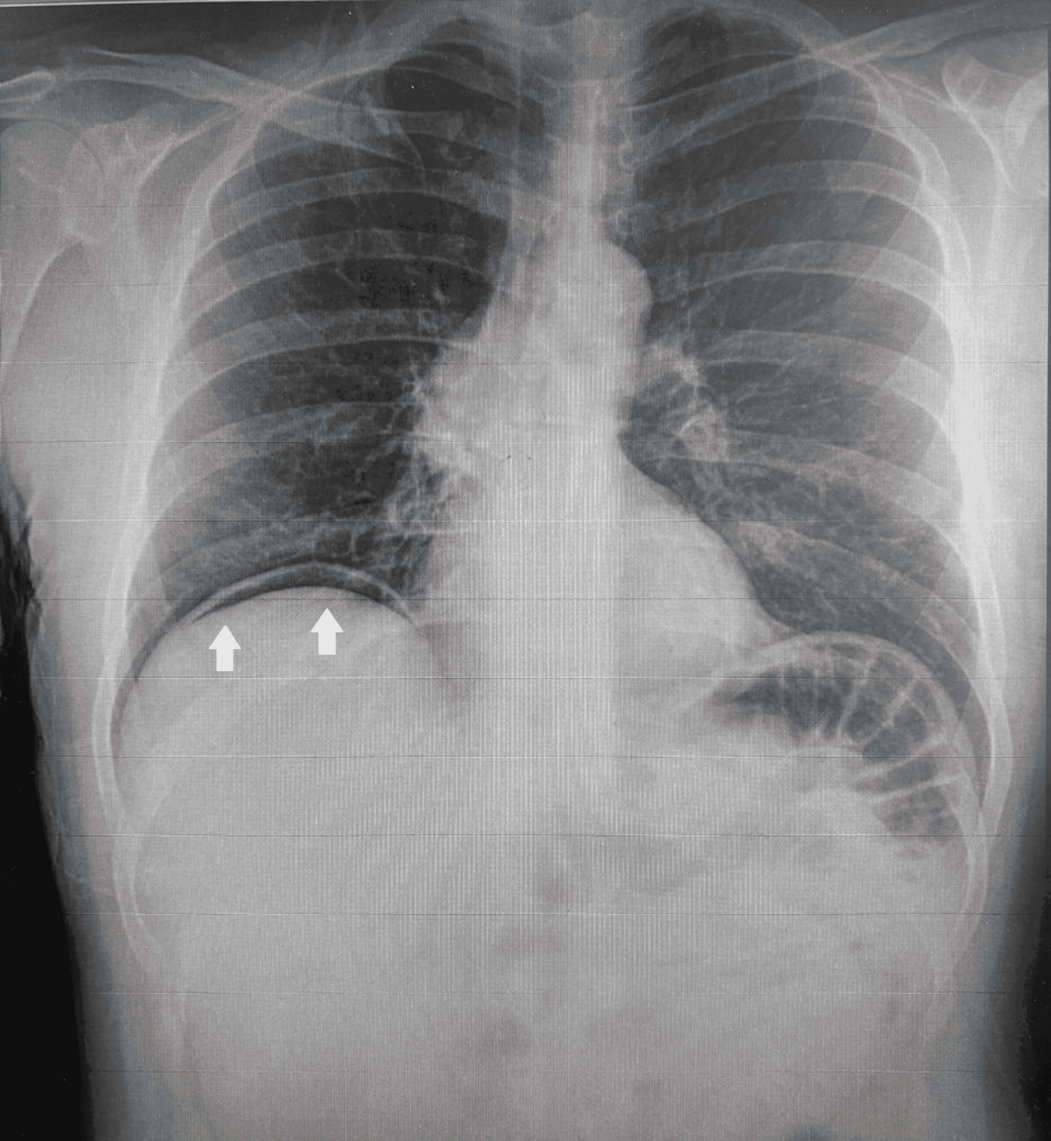
Chest X-ray showing gas under the diaphragm.

**Figure 2:**
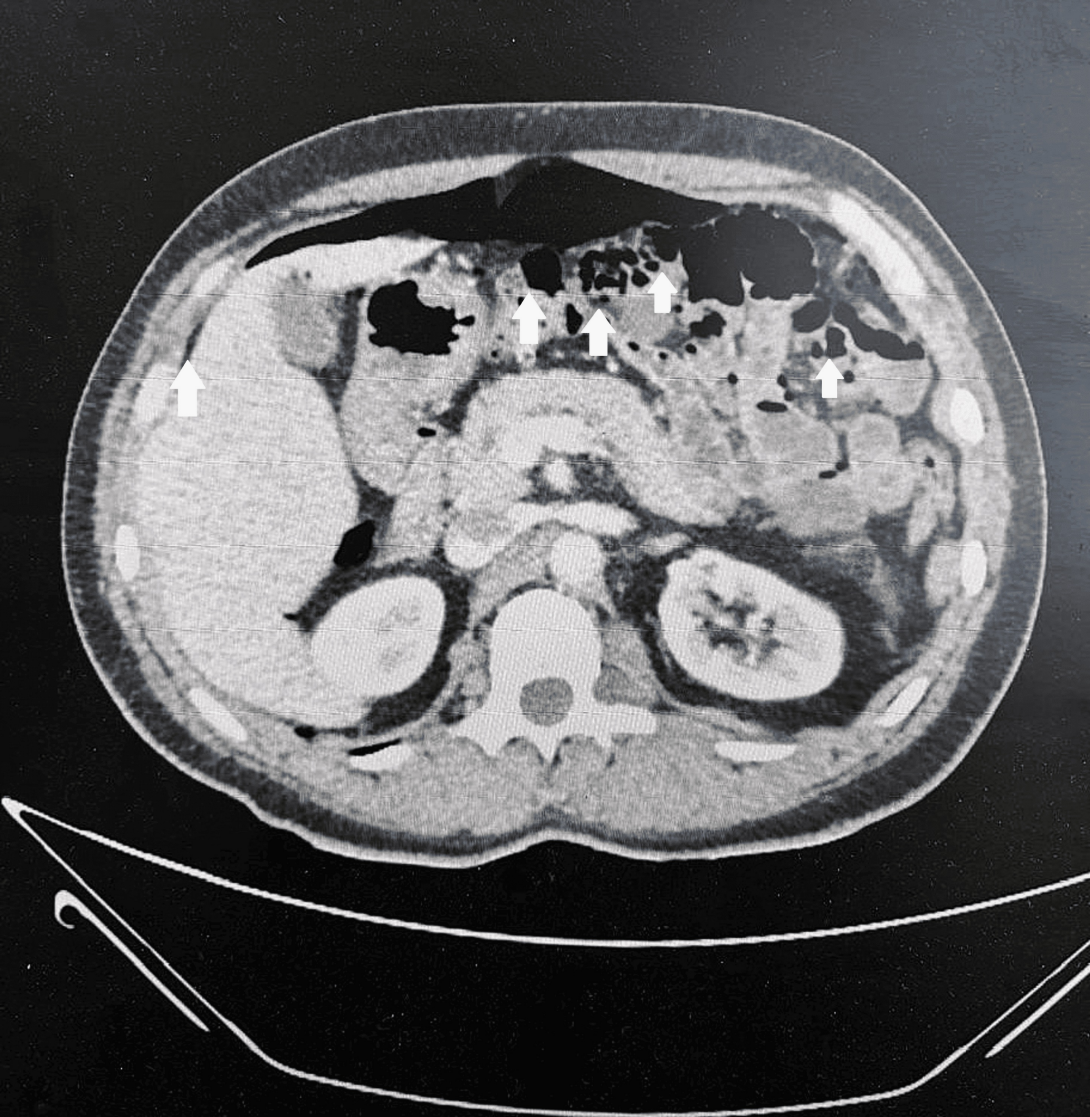
Transverse abdominal CT scan with IV contrast showing pneumoperitoneum, with multiple small bowel diverticula with pneumatosis.

**Figure 3:**
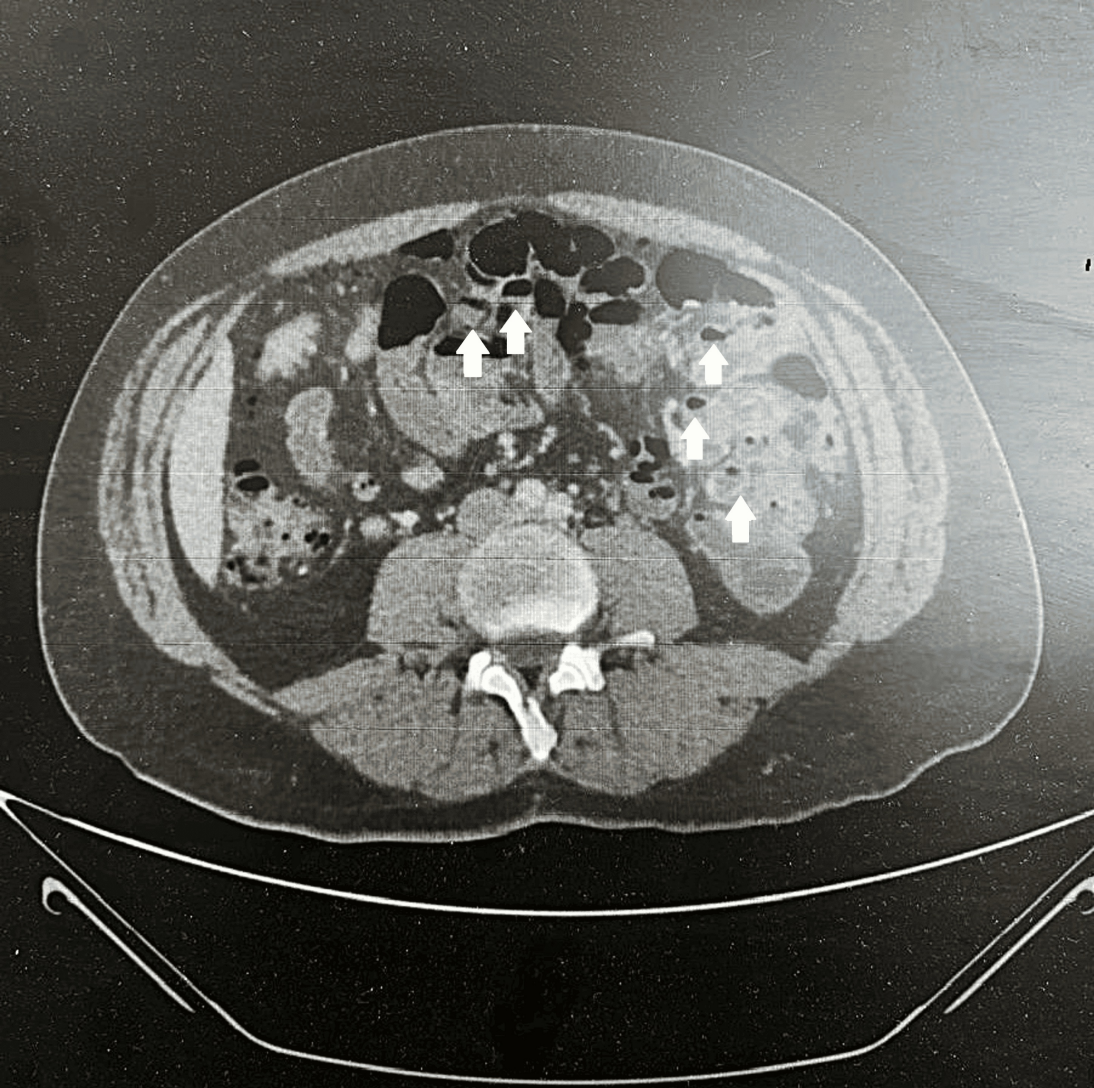
Transverse abdominal CT scan with IV contrast showing multiple small bowel diverticula with pneumatosis.

A 38-year-old man presented with a history of vague abdominal pain, colicky in nature, for three months, and the pain increased in severity and became acute within four hours prior to presentation to the emergency department. Upon examination, his vitals were stable but showed peritonism on abdominal exam. Lab tests were within the normal range (Table 1). Chest X-rays showed gas under the diaphragm, an abdominal CT scan showed multiloculated mesenteric cysts with pneumoperitoneum, and then after careful discussion and reviewing, small bowel diverticulosis was concluded (Figures 1-3).

**Table 1:** Complete blood count.

Test	Result	Units	Ref. Range
White Blood Cells	9.4	10^3/uL	4-11
Red Blood Cells	5.5	10^6/Ul	4.4-6
Hemoglobin	16	g/dL	13.5-18
Hematocrit	47.4	%	40-51
Mean Corpuscular Volume	85.6	fL	80-100
Platelet Count	322	10^3/uL	140-450
Neutrophils	5	10^3/uL	2-7
Lymphocytes	3	10^3/uL	1.5-4

**Figure 4:**
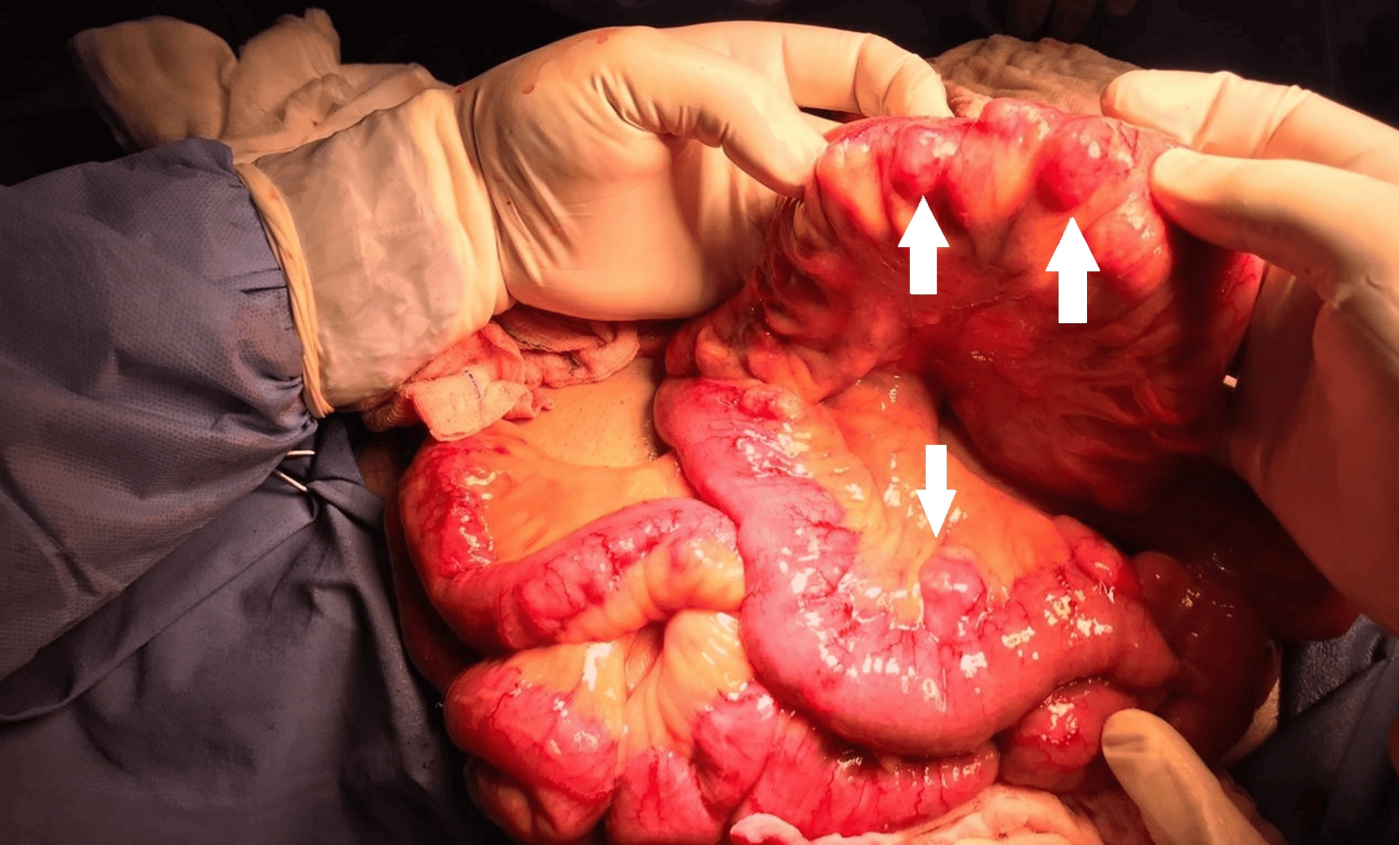
Intraoperative findings of multiple small bowel true diverticula involving the jejunum and ileum.

**Figure 5:**
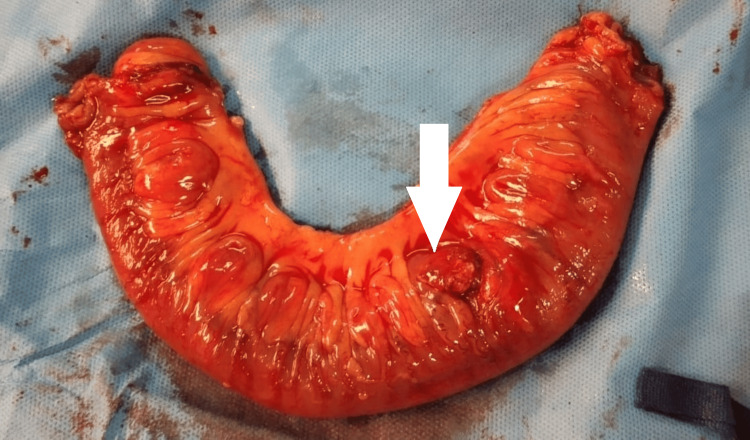
Resected specimen of 10 cm of jejunum showing perforation of the diverticula.

 The patient underwent an exploratory laparotomy, revealing multiple true diverticula in the jejunum and ileum, while the stomach and duodenum remained unaffected. A 10 cm segment of the jejunum was resected, which contained perforated diverticula (Figures 4, 5). The remaining diverticula were meticulously examined for perforation and found to be intact, so no additional intervention was performed. Histopathological analysis confirmed diverticulosis complicated by diverticulitis and perforation.

**Figure 6:**
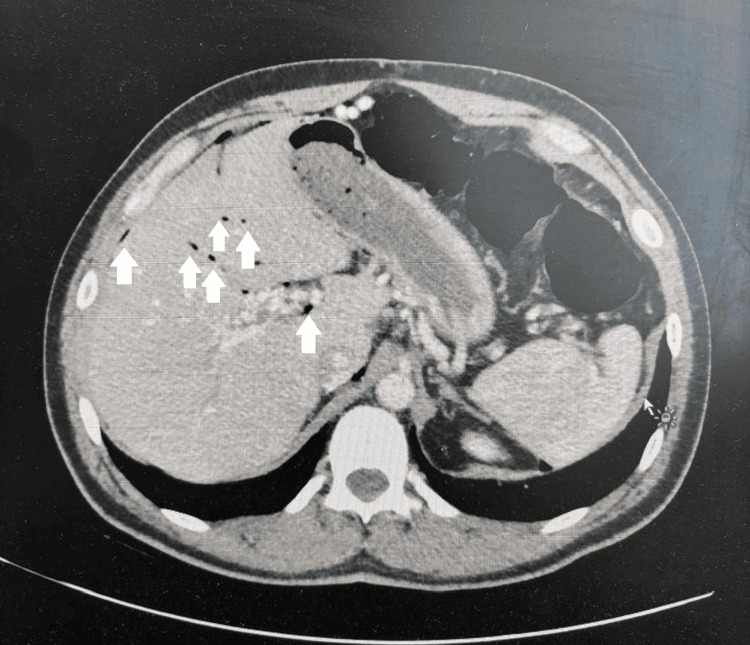
Transverse abdominal CT scan with IV contrast showing benign pneumoperitoneum.

His condition improved and was discharged home after two days with no complications. Upon follow-up for two years, the patient had multiple visits to the emergency department and the clinic for colicky abdominal pain. Although within these two years his radiological results showed pneumoperitoneum, he was treated conservatively with no surgical intervention as he did not have any signs of peritonism (Figure 6).

**Figure 7:**
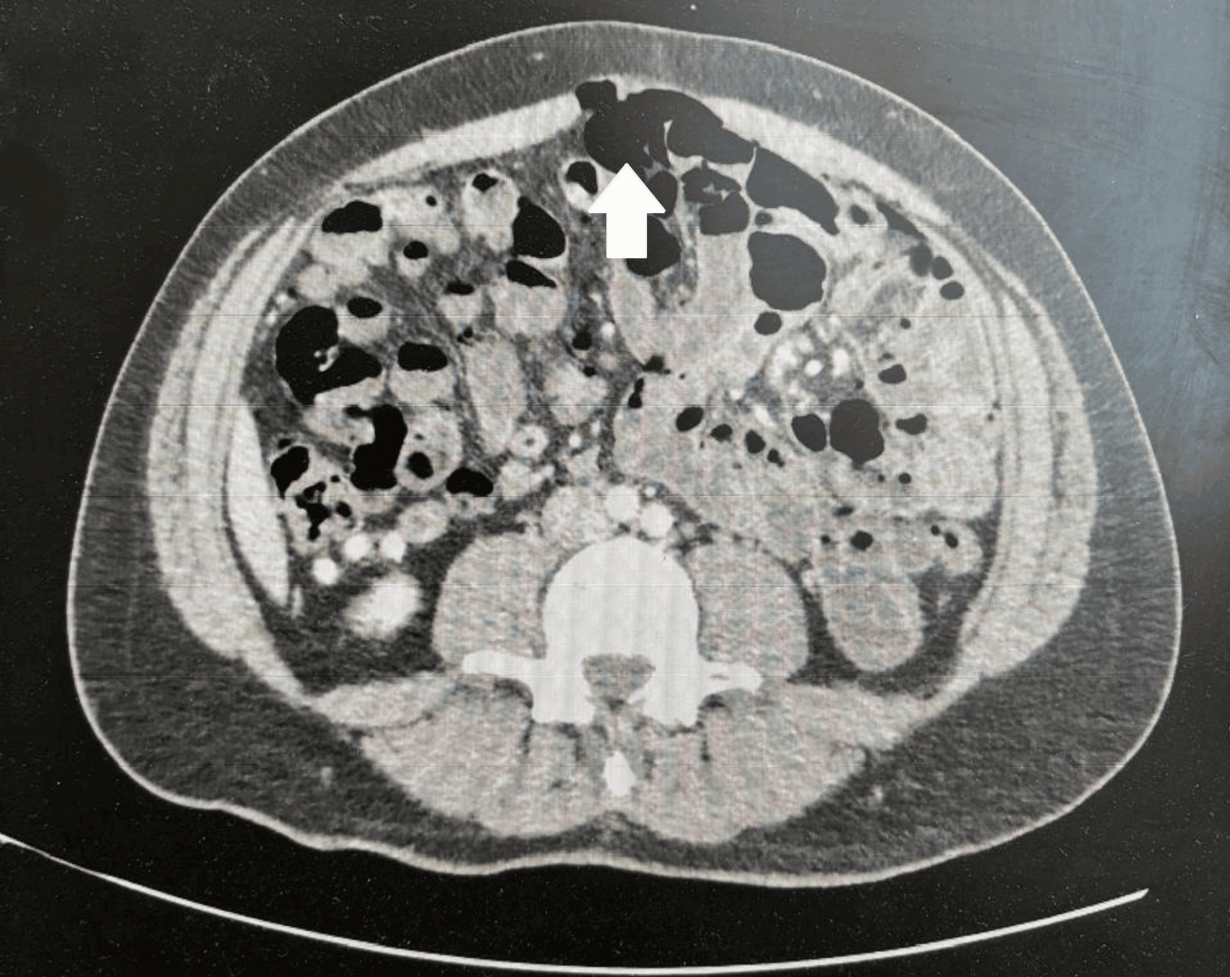
Transverse abdominal CT scan with IV contrast showed pneumoperitoneum due to perforated jejunal diverticula.

During one visit, the patient presented with severe abdominal pain, peritoneal signs, and leukocytosis (Table 2). Radiological evaluation revealed pneumoperitoneum due to perforated jejunal diverticula and multiple small bowel diverticula (Figure 7). Consequently, a second exploratory laparotomy was performed, revealing multiple adhesions from the previous surgery and perforated diverticula in the proximal jejunum. Resection of the perforated jejunal segment with anastomosis was carried out. The patient’s condition improved, and he was subsequently discharged home.

**Table 2:** Complete blood count.

Test	Result	Units	Ref. Range
White Blood Cells	14	10^3/uL	4-11
Red Blood Cells	5.16	10^6/Ul	4.4-6
Hemoglobin	14.7	g/dL	13.5-18
Hematocrit	45	%	40-51
Mean Corpuscular Volume	87.2	fL	80-100
Platelet Count	286	10^3/uL	140-450
Neutrophils	12	10^3/uL	2-7
Lymphocytes	1.6	10^3/uL	1.5-4

He continued to be followed at the clinic and was operated twice for incisional hernia and he is doing very well. Although he kept showing pneumoperitoneum on radiological images, he has been managed conservatively on follow-ups, and he will be treated conservatively as long as he does not develop any signs of peritonism.

## Discussion

The rare presentation of small bowel diverticulosis, as highlighted in this case, provides insight into the diagnostic challenges and clinical management of a condition that is often asymptomatic and may go undetected. Small bowel diverticulosis, especially in the jejunum and ileum, is uncommon, with an incidence reported to range from 0.1% to 2.3% in the general population, generally identified incidentally in imaging or during surgery [2]. This case report underscores both the clinical complexity and the diagnostic difficulties in managing small bowel diverticulosis, particularly when it leads to complications such as perforation and recurrent abdominal pain.

Diagnostic challenges in small bowel diverticulosis

The diagnostic process for small bowel diverticulosis can be challenging due to the nonspecific nature of its symptoms, which often mimic other gastrointestinal disorders. Patients may experience intermittent, nonspecific abdominal pain, as seen in this case. However, the abrupt onset of acute symptoms such as peritonism, combined with imaging findings like pneumoperitoneum, raises suspicion for complications like perforation, which can be life-threatening if not managed appropriately [4].

In this case, initial imaging with a chest X-ray showed free gas under the diaphragm, and a CT scan identified pneumoperitoneum, a classic radiological finding suggestive of perforation. Although in this case the initial CT imaging did not report diverticulosis due to its rarity, CT imaging is considered one of the most valuable diagnostic tools for small bowel diverticulosis as it can localize diverticula, identify the presence of inflammation, and detect complications such as abscess formation or perforation [3]. The chronicity of pneumoperitoneum in this patient, despite the absence of peritonism, complicates the diagnosis and raises questions about when surgical intervention is warranted.

Clinical spectrum and complications

Small bowel diverticulosis can have a wide range of clinical manifestations. While often asymptomatic, it may lead to chronic symptoms like intermittent colicky abdominal pain or complications including diverticulitis, hemorrhage, perforation, or small bowel obstruction [1]. The patient's multiple emergency visits for recurrent abdominal pain reflect the chronic, recurrent nature of symptomatic small bowel diverticulosis.

The patient’s initial laparotomy revealed true diverticula in the jejunum and ileum with a perforated diverticulum, necessitating resection. Unlike colonic diverticulosis, where diverticula are pseudo-diverticula (involving only the mucosa and submucosa), small bowel diverticula are true diverticula, which include all layers of the intestinal wall, and this makes them more prone to complications like perforation [5]. 

This patient's postoperative follow-ups showed that although the patient continued to present with pneumoperitoneum, conservative management was preferred in the absence of acute signs of peritonitis, highlighting the need for individualized management plans.

Surgical and conservative management

The management of small bowel diverticulosis is guided by the severity of symptoms and complications. This patient underwent two laparotomies for perforated jejunal diverticula, with resection and anastomosis each time, which provided symptom relief and reduced the risk of further perforations in those segments. Surgery remains the mainstay of treatment for complicated diverticulosis, especially in cases of perforation, obstruction, or recurrent diverticulitis [4]. However, conservative management may be considered for patients without signs of peritonitis or severe infection, even in cases with pneumoperitoneum, as in this patient’s follow-ups.

In chronic or recurrent cases, conservative management, including antibiotics, dietary adjustments, and symptom monitoring, is often sufficient. This approach aligns with the literature suggesting conservative treatment for small bowel diverticulosis, especially in cases where symptoms are not severe [3]. However, repeated imaging findings of pneumoperitoneum without peritonism indicate that a conservative approach may not increase morbidity, and monitoring can be a viable long-term strategy.

In the majority of cases, pneumoperitoneum is secondary to an acute visceral perforation requiring emergent surgical intervention. Chronic pneumoperitoneum, however, is less common and may be managed conservatively in selected cases if peritonitis, fever, and leukocytosis are absent [6,7]. Chiu et al. in their review of 88 patients with small bowel diverticular disease concluded that small bowel diverticulosis, with the exception of Meckel's diverticulum, did not need surgical intervention in the absence of significant symptoms [8].

In their comprehensive review, management strategies for small bowel diverticulosis, as discussed by Makris et al., are highly individualized, taking into account the patient's clinical presentation and the severity of symptoms. For asymptomatic individuals or those with mild symptoms, a conservative approach is often adopted, focusing on dietary modifications and close monitoring. In contrast, symptomatic patients, particularly those with complications like perforation or significant bleeding, may require surgical intervention. The authors caution that the extent of surgical resection should be carefully considered, especially in patients with extensive diverticulosis, to prevent short bowel syndrome [9].

Long-term outcomes and quality of life

The case highlights the potential for recurrent complications and the need for ongoing management and follow-up. Small bowel diverticulosis can impact quality of life, particularly with recurrent abdominal pain or need for surgical interventions, as seen with this patient’s repeated visits and surgeries. The development of an incisional hernia, requiring further surgical correction, also reflects a potential complication of abdominal surgeries in patients with recurrent small bowel pathology [5].

This patient’s recurrent pneumoperitoneum and lack of peritonism over multiple follow-ups indicate that not all imaging abnormalities require aggressive intervention, emphasizing a tailored approach to management based on clinical signs rather than solely on radiological findings.

## Conclusions

This case highlights the diagnostic and clinical challenges associated with small bowel diverticulosis. While often asymptomatic, small bowel diverticulosis can present with acute complications, necessitating careful evaluation and a balance between surgical and conservative management strategies. Radiological findings such as pneumoperitoneum should be interpreted in the clinical context, and conservative management may be appropriate in stable patients without peritonism. Long-term follow-up is essential for managing recurrent symptoms and complications, ultimately helping to improve patient outcomes and quality of life.
